# No association of CDK5 genetic variants with Alzheimer's disease risk

**DOI:** 10.1186/1471-2350-10-68

**Published:** 2009-07-17

**Authors:** José Luis Vázquez-Higuera, Ignacio Mateo, Pascual Sánchez-Juan, Eloy Rodríguez-Rodríguez, Jon Infante, José Berciano, Onofre Combarros

**Affiliations:** 1 Neurology Service and Centro de Investigación Biomédica en Red sobre Enfermedades Neurodegenerativas (CIBERNED), University Hospital "Marqués de Valdecilla" (University of Cantabria), Santander, Spain

## Abstract

**Background:**

As cyclin-dependent kinase 5 (CDK5) has been implicated in the abnormal hyperphosphorylation of tau in Alzheimer's disease (AD) brain, and the development of neurofibrillary tangles, we examined the contribution of this gene to the susceptibility for AD.

**Methods:**

We examined genetic variations of CDK5 by genotyping haplotype tagging SNPs (htSNPs) (rs9278, rs2069459, rs891507, rs2069454, rs1549759 and rs2069442) in a group of 408 Spanish AD cases and 444 controls.

**Results:**

There were no differences in the genotypic, allelic or haplotypic distributions between cases and controls in the overall analysis or after stratification by APOE ε4 allele.

**Conclusion:**

Our negative findings in the Spanish population argue against the hypothesis that CDK5 genetic variations are causally related to AD risk. Still, additional studies using different sets of patients and control subjects deserve further attention, since supporting evidence for association between CDK5 gene and AD risk in the Dutch population exists.

## Background

One of the neuropathological hallmarks in Alzheimer's disease (AD) is the presence of neurofibrillary tangles, which are composed of the microtubule-binding protein tau that is hyperphosphorylated [1]. Cyclin-dependent kinase 5 (CDK5) has been implicated as one of the major protein kinases involved in the abnormal hyperphosphorylation of tau in AD [2], the activity of CDK5 has been shown to be higher in the prefrontal cortex of AD brains [3], and CDK5 has been described to be associated with all stages of neurofibrillary pathology in AD brains [4]. In addition, transgenic mice overexpressing CDK5 show a dramatic increase in hyperphosphorylated, aggregated tau [5]. CDK5 is an interesting genetic target for association analysis of AD, and there are data available from 5 independent samples [6-9]. To facilitate comparisons with the Rotterdam Study that found a specific CDK5 haplotype influencing the risk for AD in a Dutch population, we examined the haplotype tagging SNPs identified by Arias-Vásquez et al. [8] (rs2069442, rs2069454, rs891507, rs2069459, and rs9278) and one additional htSNP (rs1549759), covering the complete genomic region of CDK5, in a large series of Spanish AD cases and controls.

## Methods

The study included 408 AD patients (66% women; mean age at the time of study 76.8 years; SD 6.6; range 62–97 years; mean age at onset 73.5 years; SD 6.6; range 60–93 years) who met NINCDS/ADRDA criteria for probable AD [10]. All AD cases were defined as sporadic because their family history did not mention any first-degree relative with dementia; this information was obtained by direct interview of relatives. AD patients were consecutively admitted to the Department of Neurology, University Hospital "Marqués de Valdecilla", Santander, Spain, from January 1997 to December 2001. The large majority of patients were living in the community and had been referred by their general practitioner; few had been admitted from hospital wards or nursing home facilities. Control subjects were 444 unrelated individuals (68% women; mean age 80.7 years; SD 7.6; range 62–100 years) randomly selected from a nursing home. These subjects had complete neurological and medical examinations that showed that they were free of significant illness and had Mini Mental State Examination scores of 28 or more, which were verified by at least one subsequent annual follow-up assessment. The controls arose from the same base population as the cases. The AD and control samples were Caucasians originating from a homogeneous population in a limited geographical area in Northern Spain. All patients and controls were ascertained to have parents and grandparents born in Northern Spain to ensure ethnicity. Consequently, possible confounding effects of the inclusion in the study of members of different ethnic groups have been minimized.

Blood samples were taken after written informed consent had been obtained from the subjects or their representatives. The study was approved by the ethical committee of the University Hospital "Marqués de Valdecilla". Genotyping of CDK5 (rs9278, rs2069459, rs891507, rs2069454, rs1549759, and rs2069442) polymorphism was performed by a Taq-Man single-nucleotide-polymorphism assay (Applied Biosystems, Warrington, Cheshire, UK) and an ABI PRISM 7000 sequence detection system (Applied Biosystems). APOE genotyping was performed by amplification of the 4th exon of the APOE gene by PCR with biotinylated primers, followed by reverse hybridization on nitrocellulose strips, using the INNO-LIPA ApoE assay (Innogenetics NV, Ghent, Belgium).

Hardy-Weinberg equilibrium (HWE) was calculated for the 6 htSNPs genotypes in the control population using Pearson's χ^2 ^statistics. We assessed pairwise linkage disequilibrium (LD) between the 6 htSNPs by D' and r^2 ^statistics. Haplotype reconstruction and their frequencies in cases and controls were estimated by an expectation-maximization algorithm. Pearson's χ^2 ^statistics were performed to compare allele distribution of the patients and control for each htSNP. Haplotype frequencies were also assessed using Pearson's χ^2 ^using Haploview 3.32 software . Rare haplotypes (total frequency < 0.05) were excluded from the analysis.

## Results

In control groups, no deviations from Hardy-Weinberg equilibrium were found for any of the 6 htSNPs (p values ranging from 0.15 to 0.71). As shown in Table 1, the distribution of the allele and genotype frequencies of the CDK5 htSNPs did not differ significantly between either un-stratified or APOE-stratified AD and control groups. Haplotype distributions were not significantly different between cases and controls in the overall analysis or after stratification by APOE ε4 allele (Table 2). There were no major differences in allele, genotype or haplotype frequencies of CDK5 polymorphisms in our total sample associated to either age or gender subgroups (data not shown).

**Table 1:** Distribution of CDK5 polymorphisms in patients and controls stratified by APOE ε4 allele

CDK5 polymorphism		APOE ε4 allele noncarriers		APOE ε4 allele carriers		Total sample	
				
		Patients	Controls	Patients	Controls	Patients	Controls
rs9278	GG	125 (0.67)	259 (0.71)	145 (0.66)	55 (0.71)	270 (0.66)	314 (0.71)
	GA	61 (0.33)	96 (0.26)	69 (0.31)	22 (0.28)	130 (0.32)	118 (0.27)
	AA	1 (0.00)	10 (0.03)	7 (0.03)	1 (0.01)	8 (0.02)	11 (0.02)
	Total	187	365	221	78	408	443
	Allele frequency G/A	0.83/0.17	0.84/0.16	0.81/0.19	0.85/0.15	0.82/0.18	0.84/0.16
rs2069459	GG	60 (0.34)	116 (0.32)	74 (0.35)	23 (0.29)	134 (0.34)	139 (0.31)
	GT	83 (0.47)	169 (0.46)	100 (0.47)	39 (0.50)	183 (0.47)	208 (0.47)
	TT	33 (0.19)	81 (0.22)	39 (0.18)	16 (0.21)	72 (0.19)	97 (0.22)
	Total	176	366	213	78	389	444
	Allele frequency G/T	0.58/0.42	0.55/0.45	0.58/0.42	0.54/0.46	0.58/0.42	0.55/0.45
rs891507	GG	101 (0.58)	211 (0.58)	123 (0.60)	48 (0.64)	224 (0.59)	259 (0.59)
	GA	60 (0.35)	135 (0.37)	73 (0.36)	24 (0.32)	133 (0.35)	159 (0.36)
	AA	12 (0.07)	16 (0.05)	9 (0.04)	3 (0.04)	21 (0.06)	19 (0.05)
	Total	173	362	205	75	378	437
	Allele frequency G/A	0.76/0.24	0.77/0.23	0.78/0.22	0.80/0.20	0.77/0.23	0.77/0.23
rs2069454	GG	171 (0.97)	320 (0.91)	199 (0.93)	68 (0.91)	370 (0.94)	388 (0.91)
	GC	6 (0.03)	30 (0.09)	16 (0.07)	7 (0.09)	22 (0.06)	37 (0.09)
	CC	0 (0.00)	0 (0.00)	0 (0.00)	0 (0.00)	0 (0.00)	0 (0.00)
	Total	177	350	215	75	392	425
	Allele frequency G/C	0.98/0.02	0.96/0.04	0.96/0.04	0.95/0.05	0.97/0.03	0.96/0.04
rs1549759	GG	112 (0.69)	244 (0.70)	134 (0.67)	57 (0.74)	246 (0.68)	301 (0.70)
	GA	48 (0.30)	100 (0.28)	59 (0.30)	20 (0.26)	107 (0.30)	120 (0.28)
	AA	2 (0.01)	7 (0.02)	6 (0.03)	0 (0.00)	8 (0.02)	7 (0.02)
	Total	162	351	199	77	361	428
	Allele frequency G/A	0.84/0.16	0.84/0.16	0.82/0.18	0.87/0.13	0.83/0.17	0.84/0.16
rs2069442	CC	93 (0.55)	204 (0.60)	127 (0.63)	40 (0.57)	220 (0.60)	244 (0.59)
	CG	65 (0.39)	121 (0.35)	60 (0.30)	25 (0.36)	125 (0.34)	146 (0.35)
	GG	10 (0.06)	18 (0.05)	14 (0.07)	5 (0.07)	24 (0.06)	23 (0.06)
	Total	168	343	201	70	369	413
	Allele frequency C/G	0.75/0.25	0.76/0.24	0.78/0.22	0.75/0.25	0.77/0.23	0.77/0.23

Figures in parentheses indicate frequencies; p-values > 0.05 for all allelic and genotypic comparisons; p-values were not corrected for multiple comparisons.

**Table 2:** Haplotype association analysis between CDK5 gene and AD stratified by APOE ε4 allele

CDK5 haplotype	APOE e4 allele noncarriers		APOE e4 allele carriers		Total sample	
			
	AD, control frequency	p-value	AD, control frequency	p-value	AD, control frequency	p-value
GTGGGC	0.29, 0.31	0.30	0.29, 0.33	0.36	0.29, 0.31	0.30
GGGGGG	0.16, 0.17	0.59	0.14, 0.18	0.21	0.16, 0.17	0.59
GGGGAC	0.14, 0.12	0.16	0.15, 0.09	0.07	0.14, 0.12	0.16
AGGGGC	0.12, 0.11	0.57	0.14, 0.14	0.94	0.12, 0.11	0.57
GTAGGC	0.09, 0.10	0.74	0.07, 0.08	0.91	0.09, 0.10	0.74
GGAGGG	0.06, 0.05	0.53	0.06, 0.05	0.66	0.06, 0.05	0.53

Haplotype block consists of SNPs: rs9278, rs2069459, rs891507, rs2069454, rs1549759 and rs2069442. Rare haplotypes (total frequency < 0.05) were excluded from the analysis. P-values were not corrected for multiple comparisons.

## Discussion

In a series of 85 early onset AD patients from Sweden and 104 early onset patients from the Netherlands, Rademakers et al. [6] studied five tagging SNPs (rs2069442, rs2069454, rs891507, rs2069459, and rs9278), observing a two times increased AD risk in both patient samples for carriers of the CDK5 rs2069454 C allele. Assessing the association between these previously described htSNPs in the CDK5 gene and late onset AD in a Dutch case-control series, Arias-Vásquez et al. [8] observed a 1.8-fold (p = 0.001) increased risk of AD for carriers of the GG genotype of SNP rs2069442 in the CDK5 gene in non-carriers of the APOE ε4 allele; in addition the CDK5 haplotype (GGGGG) composed of 5'UTR (rs2069442), intron 5 (rs2069454), intron 9 (rs891507 and rs2069459) and 3'UTR (rs9278) was significantly associated with AD (OR = 1.21, p = 0.05) in non-carriers of the APOE ε4 allele. We genotyped the same variants in CDK5 as described by Arias-Vásquez et al, but we were not able to confirm these results, although we had enough power (80%) to detect an OR as low as 1.43 for an increased risk effect of CDK5 haplotype in APOE ε4 allele non-carriers. Our failure to replicate the main finding of Arias-Vásquez et al. could be caused by many factors. We might have not been able to detect the effect due to the fact that in the original study the GGGGG haplotype conferred only a modest risk (OR = 1.2), and the possibility of a type 2 error (false-negative) must be taken into account. Another possibility is genetic heterogeneity between our sample sets and those of the original study. In two independent studies conducted in USA and Netherlands [7], and Canada [9], respectively, the CDK5 rs2069454 and rs891507 genetic variants were not associated with AD. AlzGene database  reported meta-analyses of these two CDK5 polymorphisms, both of which are studied here, without any significant effects (based on nearly 10,000 cases and controls) [11].

## Conclusion

Our negative findings in the Spanish population argue against the hypothesis that CDK5 genetic variations are causally related to AD risk. Still, additional studies using different sets of patients and control subjects deserve further attention, since supporting evidence for association between CDK5 gene and AD risk in the Dutch population exists.

## Competing interests

The authors declare that they have no competing interests.

## Authors' contributions

JLVH and ERR performed the genetic studies and reviewed critically the manuscript. PSJ performed the statistical analyses and reviewed critically the manuscript. IM, JI and JB reviewed critically the manuscript. OC drafted the manuscript and contributed to its final version. All authors read and approved the final manuscript.

## Pre-publication history

The pre-publication history for this paper can be accessed here:


